# Supplementary material for: Monitoring ocean currents during the passage of Typhoon Muifa using optical-fiber distributed acoustic sensing
Source: Nat Commun. 2024 Feb 6;15:1111. doi: 10.1038/s41467-024-45412-x (PMC10847141; doi:10.1038/s41467-024-45412-x)
Supplement: Supplementary file 1 — Supplementary Information [file 41467_2024_45412_MOESM1_ESM.pdf]

Supplementary Information

Monitoring ocean currents during the passage of Typhoon Muifa using optical-fiber distributed acoustic sensing

Jianmin Lin[*1,2], Sunke Fang[1], Runjing He[1], Qunshu Tang[1,2], Fengzhong Qu[1,2], Baoshan Wang[*3,4] and Wen Xu[*1,2]

[1]Key Laboratory of Ocean Observation-Imaging Testbed of Zhejiang Province, Zhejiang University, Zhoushan, 316021, China

[2]Donghai Laboratory, Zhoushan, 316021, China

[3]Deep Space Exploration Laboratory/School of Earth and Space Sciences, University of Science and Technology of China, Hefei, 230026, China

[4]Mengcheng National Geophysical Observatory, University of Science and Technology of China, Hefei, 230026, China

Correspondence to: J. Lin, jmlin@zju.edu.cn; B. Wang, bwgeo@ustc.edu.cn and W. Xu, wxu@zju.edu.cn

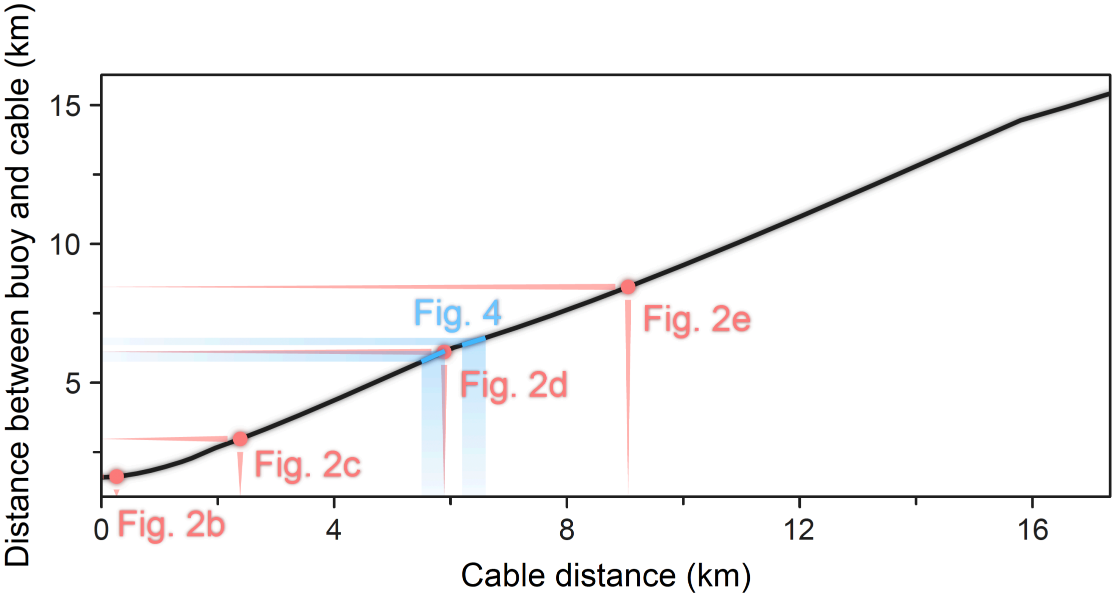

Supplementary Fig. 1. Distance between the ocean buoy and the submarine cable. The four coral dots mark out the distances between the buoy and the four selected channel segments in Fig. 2b-e of the main text respectively. The two blue line segments denote the distance ranges between the buoy and the selected cable segments in Fig. 4 of the main text.

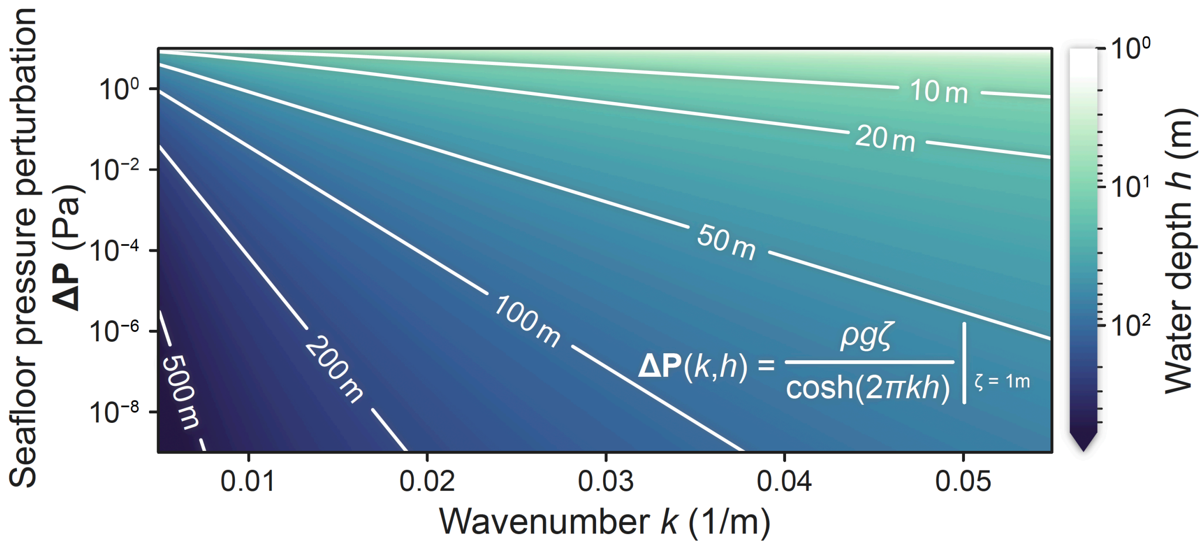

Supplementary Fig. 2. Decrease of seafloor pressure perturbation $\Delta \mathrm{P}(k,h) = \frac{\rho g \zeta}{\cosh(2\pi k h)}$ induced by an ocean surface gravity wave of height $\zeta = 1$ m with water depth h and wavenumber k. The colormap denotes the water depth range. The white curves represent the contours of **ΔP** when h = 10, 20, 50, 100, 200 and 500 m, respectively.

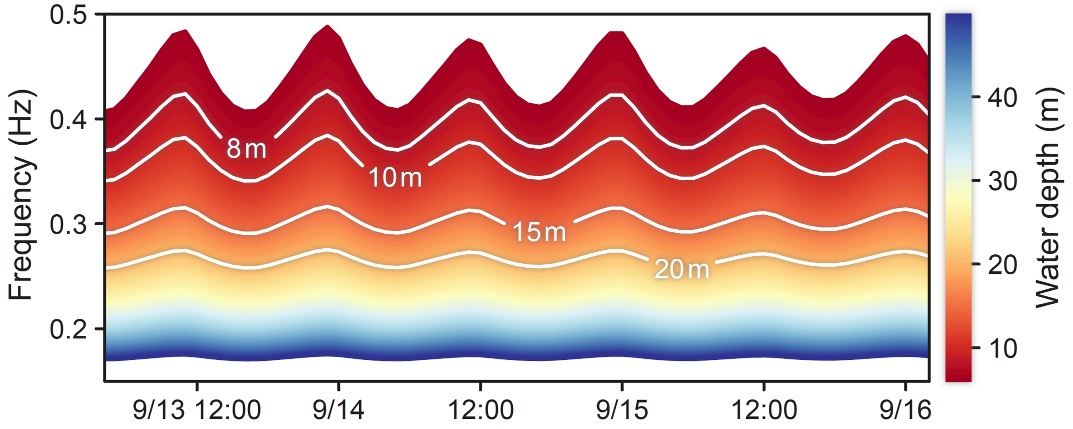

Supplementary Fig. 3. Variations of theoretical maximum frequency of the seafloor pressure induced by ocean surface gravity wave $\mathbf{f_{max}}(t)$ under different tide-modulated water depths. The colormap corresponds to water depth at low-tide time $h_{low-tide}$, and the tide-modulated water depth is derived by simply adding $h_{low-tide}$ and tide level up. The four white curves represent the $\mathbf{f_{max}}(t)$ when $h_{low-tide}$ = 8, 10, 15 and 20 m, respectively.

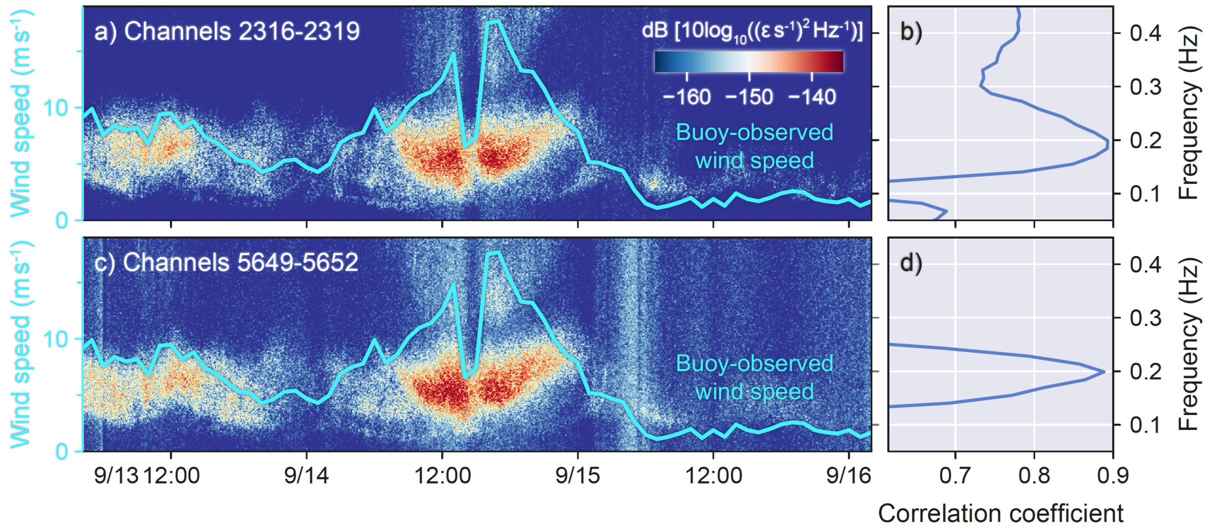

Supplementary Fig. 4. Correlation between spectrograms recorded by the distributed acoustic sensing observatory and buoy-observed wind speed. **a**, **c** Mean spectrogram at channels 2316–2319 and 5649–5652 respectively. The solid cyan line denotes the wind speed observed by the collocated ocean buoy. **b**, **d** Correlation coefficients between the mean spectrogram and observed wind speed at different frequencies. The correlation coefficients $\mathbf{R}(f)$ between spectrogram **S** and buoy-observed surface wind speed **W** was calculated as $\mathbf{R}(f) = \text{corroef}[\mathbf{S}(f), \mathbf{W}]$, where f is the frequency and corroef is a function of Python package Numpy for correlation coefficients calculation. Then the maximum Pearson correlation coefficients R_{max} and corresponding frequency f_R is derived by $\begin{cases} R_{max} = \max(\mathbf{R}(f)) \\ f_R = \text{argmax}(\mathbf{R}(f)) \end{cases}$.

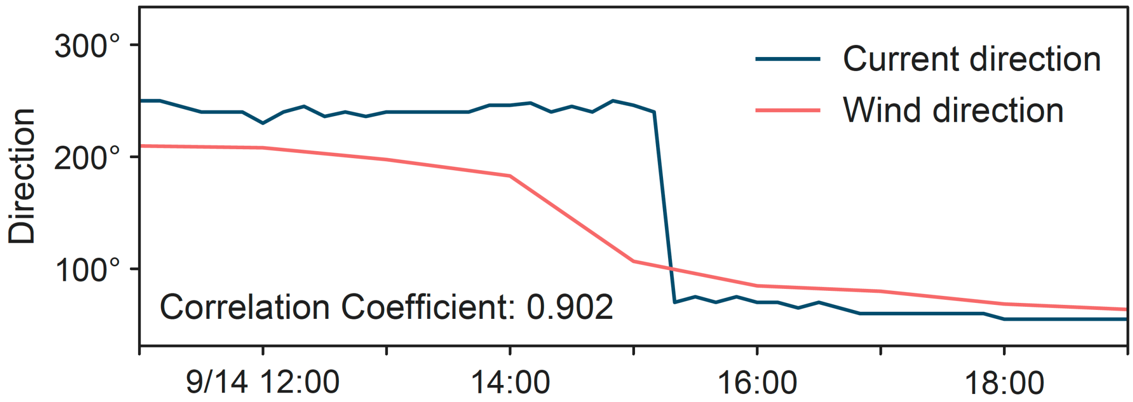

Supplementary Fig. 5. Correlation of measured ocean current directions (solid dark blue line) and buoy-observed wind directions (solid coral line). The correlation coefficient R between measured ocean current directions $\mathbf{C_{dir}}$ and buoy-observed surface wind directions $\mathbf{W_{dir}}$ was calculated as $R = \mathrm{corroef}[\mathbf{C_{dir}}, \mathbf{W_{dir}}]$, where corroef is a function of Python package Numpy for correlation coefficients calculation.

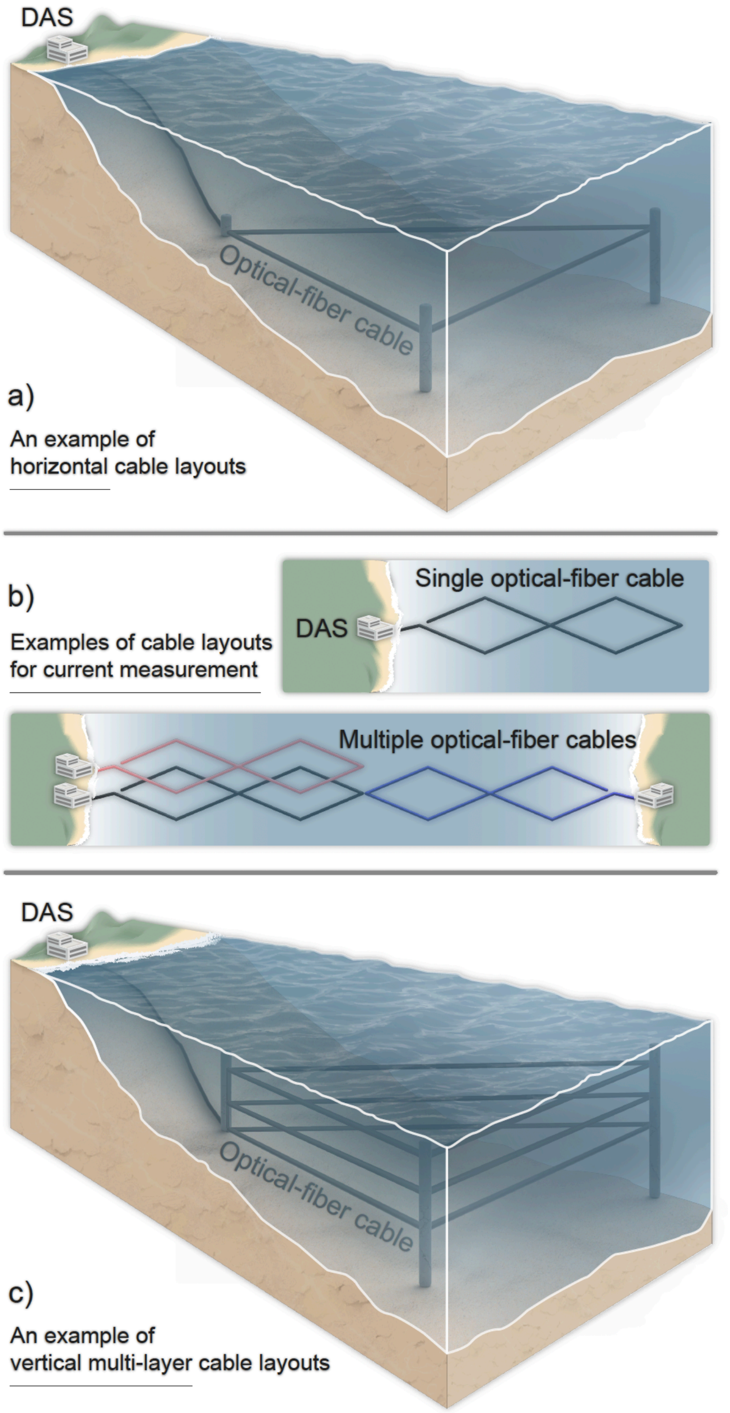

Supplementary Fig. 6. Schematic illustration of the cable configuration aimed for measuring ocean currents using distributed acoustic sensing (DAS). **a** Horizontal arrangement of cable segments designed for mitigating the impact of inconstant water depth. **b** Schemes of cable layouts designed to improve the spatial coverage and resolution of ocean current measurements. **c** Schemes of cable layouts designed to retrieve the vertical variations of horizontal ocean currents.

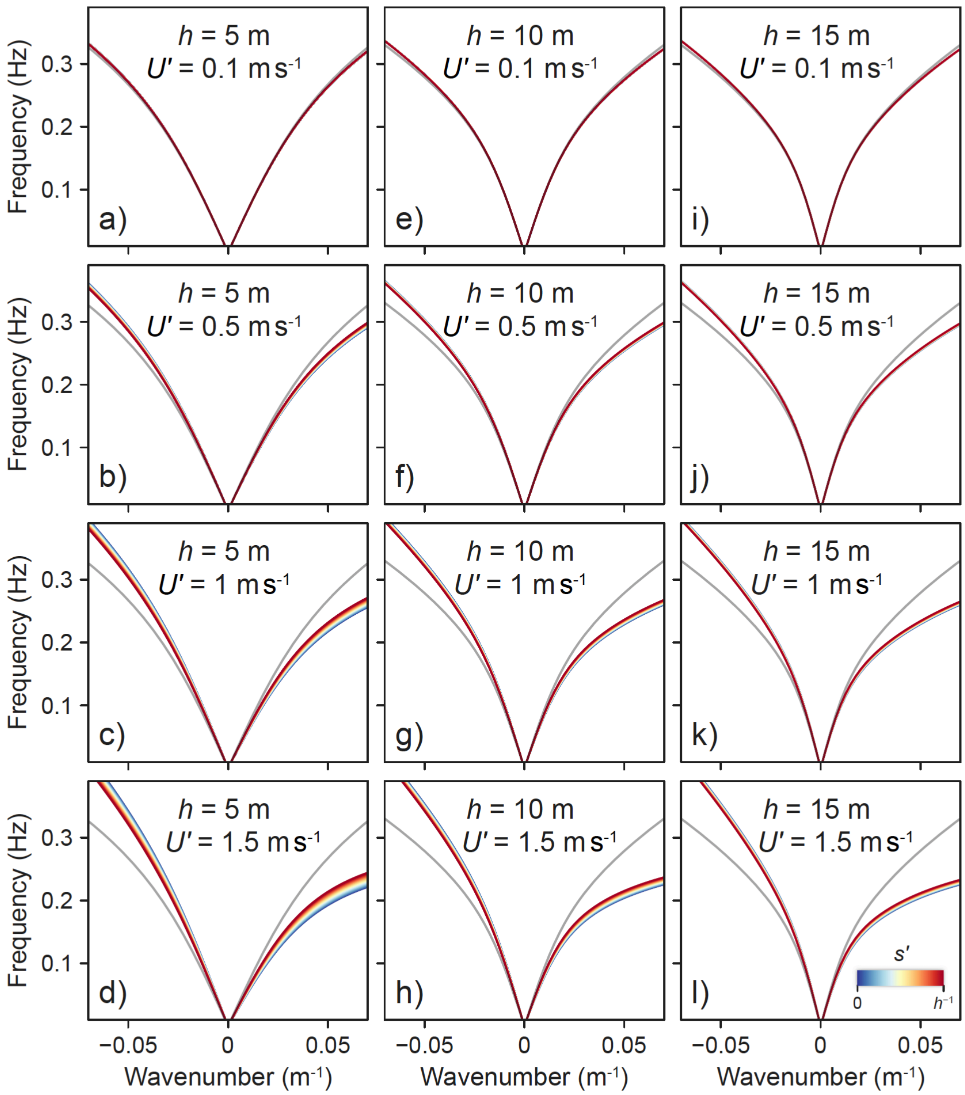

Supplementary Fig. 7. Comparison of linear ocean surface gravity wave (OSGW) dispersion curves with Doppler shifts caused by linear shear currents under different water depths h and surface current speeds U'. The Doppler-shifted OSGW dispersion curves is expressed as $\left(f+\left(1-\frac{s'\tanh(2\pi kh)}{4\pi k}\right)U'k\right)^2=\frac{gk}{2\pi}\cdot\tanh(2\pi kh)$, where f is the frequency, k is the wavenumber, $s'\in[0,h^{-1}]$ is the unknown constant gradient and $\left(1-\frac{s'\tanh(2\pi kh)}{4\pi k}\right)U'k$ is the Doppler shift term[1]. The distribution of the possible Doppler-shifted OSGW dispersion curves depends on the weighting factor of the Doppler shift term $\left(1-\frac{s'\tanh(2\pi kh)}{4\pi k}\right)$ and is shown as rainbow-colored band. The Doppler shift terms induced by linear shear currents flowing across the two adjacent cable segments with distinct water depths could be significantly different, especially in oceanic settings characterized by shallow bathymetry and rapid surface currents. This would affect the fitting progress and result in biased measurement of current speeds and directions using our method. For comparison, the corresponding linear OSGW dispersion curves are superimposed as gray curves.

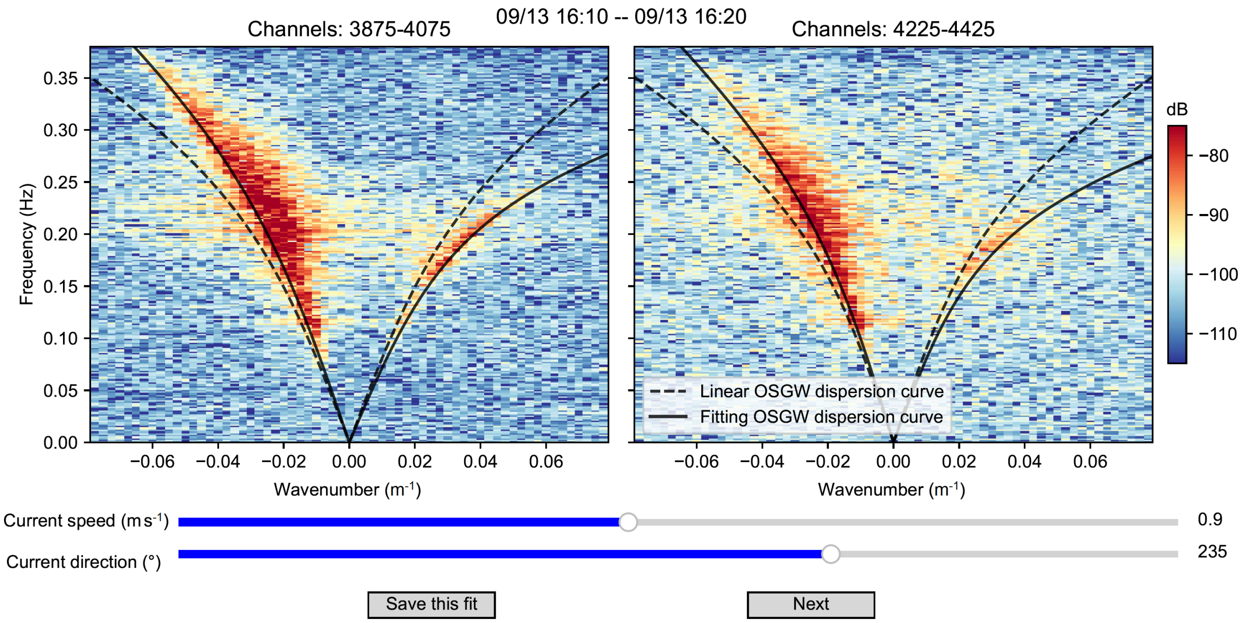

Supplementary Fig. 8. Graphical user interface (GUI) for manual fitting process of the current-induced Doppler shift. The dashed black curves represent the linear ocean surface gravity wave (OSGW) dispersion curves for channels 3875–4075 and 4225–4425. The solid black curves represent the Doppler-shifted OSGW dispersion curves, changing with the values of current speed U and direction θ, which are controlled by the white handles on the two sliders respectively. The fitting process is conducted by manually sliding the handles to synchronously fit the Doppler-shifted OSGW dispersion curves to the lower edges of dominant spectral energy packets in the frequency-wavenumber spectra of both cable segments. The estimated values of current speed U and direction θ can be saved by clicking the "Save this fit" button. The "Next" button is used for the fitting process of the next spectra pair.

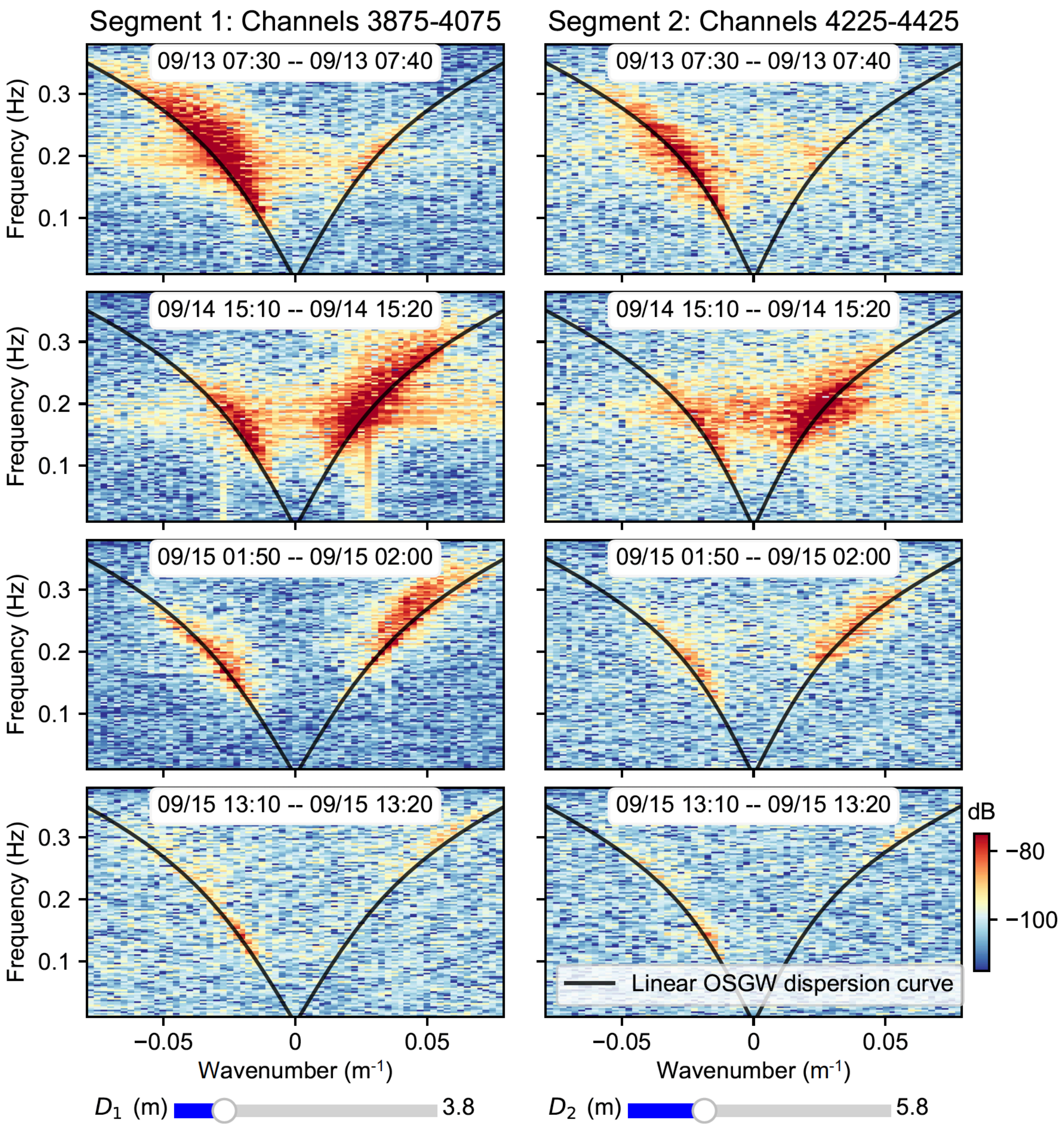

Supplementary Fig. 9. Graphical user interface (GUI) for estimation of the characteristic water depths of cable segments. The frequency-wavenumber ($f-k$) spectra of channels 3875–4075 and 4225–4425 with inconspicuous Doppler shifts at four different time periods: 07:30–07:40 UTC on September 13, 15:10–15:20 UTC on September 14, 01:50–02:00 UTC on September 15 and 13:10–13:20 UTC on September 15 are used for estimation. The black curves represent the linear ocean surface gravity wave (OSGW) dispersion curves for the two cable segments, changing with the values of the tide-modulated water depths $\mathbf{h}'_{1,2}(t)$. The characteristic water depths D_1 and D_2 are estimated by manually sliding the white handles on the "D_1" and "D_2" Sliders to optimize the fit between the corresponding linear OSGW dispersion curves and the lower edges of dominant spectral energy packets in all the $f-k$ spectra.

Supplementary References

1. Williams, E. F. *et al*. Surface Gravity wave interferometry and ocean current monitoring with ocean-bottom DAS. *J. Geophys. Res. Oceans* **127**(5), e2021JC018375 (2022).
