# Supplementary material for: Monitoring ocean currents during the passage of Typhoon Muifa using optical-fiber distributed acoustic sensing
Source: Nat Commun. 2024 Feb 6;15:1111. doi: 10.1038/s41467-024-45412-x (PMC10847141; doi:10.1038/s41467-024-45412-x)
Supplement: Supplementary file 3 — Description of Additional Supplementary Files [file 41467_2024_45412_MOESM3_ESM.pdf]

Description of Additional Supplementary Files

File Name: Supplementary Movie 1

Description: This MP4 file is a Supplementary Movie of the stepwise ocean surface gravity wave (OSGW) dispersion curve fit and corresponding ocean-current measurements. The frequency-wavenumber spectra in each frame are calculated using the 10-min observations along **a** segment 1 and **b** segment 2 in Fig. 4. The overlaid dashed black lines represent the linear OSGW dispersion curve, and the solid black lines denote the dispersion curves under the influence of an ocean current. **c** The measured current speed and direction are represented by the blue arrow, while the pale gray arrows denote the historical current measurements. **d** The red and blue dots represent the tide-modulated water depths of segments 1 and 2 used for current measurements, respectively, and corresponding curves denote the historical water depths.
